# Acute Brucellosis Presenting as an Autoimmune Hemolytic Anemia

**DOI:** 10.1155/2018/1030382

**Published:** 2018-10-25

**Authors:** Durga Shankar Meena, Vikram Singh Sonwal, Amit Kumar Rohila, Vasudha Meena

**Affiliations:** ^1^Department of General Medicine, All India Institute of Medical Sciences, Jodhpur, India; ^2^Department of Pediatrics, SN Medical College, Jodhpur, India

## Abstract

Brucellosis is one of the most widespread zoonosis in the world. Hematological complications in brucellosis usually present as mild anemia, leukopenia, or pancytopenia. Autoimmune hemolytic anemia in brucellosis is rarely reported. Here, we report an 18-year-old female presented to us with progressive fatigue, jaundice, and fever. Hematological investigations revealed hemolytic anemia. Direct Coombs test was positive. Further evaluation showed positive serology and culture for Brucella. The patient was diagnosed with brucellosis with autoimmune hemolytic anemia. She was put on rifampicin and doxycycline along with corticosteroids. After 6 weeks, the patient was symptomatically improved with complete remission of hemolytic anemia. The possibility of brucellosis should be considered as a differential diagnosis of autoimmune hemolytic anemia, especially those living in the endemic areas.

## 1. Introduction

Brucellosis is a significant public health problem in developing countries like India, where most of the rural population lives in close contact with animals. The worldwide annual occurrence of brucellosis is more than 500,000 cases [1]. The clinical spectrum of brucellosis is variable, ranging from asymptomatic disease to severe fatal illness. Musculoskeletal involvement is the most common presentation. Anemia, leukopenia, and pancytopenia are common hematological manifestations described in brucellosis. In the literature, there are few case reports describing autoimmune hemolytic anemia as a presenting feature of brucellosis; hence, we are describing an 18-year-old female of brucellosis presenting with Coombs-positive hemolytic anemia.

## 2. Case Report

An 18-year-old female presented to our hospital with chief complaints of progressive fatigue, fever, myalgia, and shortness of breath for last 3 weeks. There was no significant past illness. There was no history of significant weight loss, cough, orthopnea, pain abdomen. On physical examination, she was febrile, pale, and icteric. The spleen was palpable 2 cm below the left costal margin. Her pulse rate was 102/minute with a blood pressure of 106/70 mmHg.

**Figure 1:**
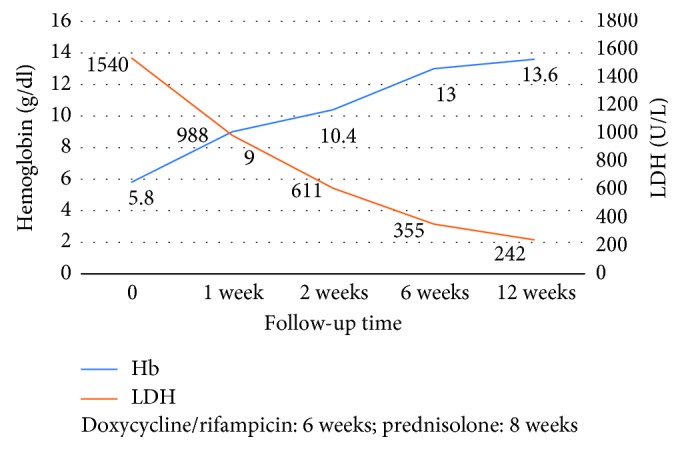
Patient laboratory values at admission and at treatment conclusion.

A complete blood count (CBC) revealed severe anemia (hemoglobin—5.8 g/dl, mean corpuscular volume (MCV)—92 fl) with a platelet count of 148 × 10^3^/*µ*L and white blood cell count (WBC) of 3.37 × 10^3^/*µ*L. A peripheral blood smear showed few spherocytes, nucleated red blood cells. Biochemistry showed indirect hyperbilirubinemia with high lactate dehydrogenase (LDH—1540 IU/L). On further investigations, corrected reticulocyte count was 5.4%. A direct Coombs test was strongly positive (4+). Based on initial investigations, we made an initial diagnosis of autoimmune hemolytic anemia. Viral markers (HIV, HBs Ag, anti-HCV) were negative. Serology for Epstein–Barr virus (EBV) and mycoplasma was also negative. Antinuclear antibodies were absent. Our patient remained febrile during hospitalisation, which was not explained by hemolytic anemia; on further evaluation, there was a recent history of consumption of unpasteurized milk. Since Brucella is one of the common zoonotic diseases in western India, we suspected brucellosis. The serology for brucellosis was positive in high titre (standard agglutination test—1:640). The diagnosis was confirmed with positive blood culture for *Brucella melitensis*. We made a final diagnosis of acute brucellosis with Coombs-positive hemolytic anemia. The patient was prescribed a combination of oral doxycycline (100 mg twice a day) with rifampicin (600 mg once a day). She was also prescribed corticosteroids (prednisolone 1 mg/kg/day). 1 week after starting steroids, the patient showed significant clinical improvement with a hemoglobin count of 9 gm/dl and serum LDH of 988 IU/L. The patient was discharged, and steroid was gradually tapered with doxycycline, and rifampicin was advised for further 5 weeks. After 6 weeks, corticosteroid was tapered successfully. The patient was symptomatically better with a hemoglobin count of 13 gm/dl (Figure 1). She was doing well with complete remission of hemolytic anemia at 3-month follow-up.

## 3. Discussion

Brucellosis is a zoonotic infection, transmitted from animal to human by direct contact or consumption of unpasteurized milk, dairy products. Brucella are small Gram-negative intracellular coccobacilli. Most of the cases with brucellosis are attributed to *Brucella melitensis*.

Brucellosis usually presents with acute febrile illness and musculoskeletal symptoms. Hematological complications are common in brucellosis which includes anemia, leukopenia, or pancytopenia. Brucella organism shows high affinity for the reticuloendothelial system and bone marrow. Anemia is mild, normocytic normochromic, attributed to transient bone marrow suppression and hypersplenism [2]. Immune thrombocytopenic purpura is also reported in brucellosis [3, 4]. Disseminated intravascular coagulation is the possible mechanism causing thrombocytopenia in one report [5]. Bone marrow suppression is usually transient, which is reversible with prompt antibiotic therapy. Hemolysis in brucellosis is rarely described in the literature with microangiopathic hemolytic anemia (MAHA) is the most common presentation [6]. The first case report of autoimmune hemolytic anemia in brucellosis was described due to cold agglutinin antibodies [7], but hemolysis was mild in their case which was resolved with antibiotic treatment for brucellosis. However, our literature review revealed few case reports associated with severe hemolysis in brucellosis (Table 1) [8–11]. Similar to these reports, our patient had severe hemolysis which required immunosuppressive treatment. Brucella can induce a systemic autoimmune response. Molecular mimicry in Brucella infection can lead to severe hemolysis and thrombocytopenia. One report described the possible role of cross-reactive antibodies to Brucella causing agglutination and hemolysis of RBC [7]. According to a recent hypothesis, multiple passages of Brucella strain in vitro can increase the expression of hemolysin gene, which might explain their pathogenicity and relation with hemolytic anemia [12].

Although hemolysis was severe, still none of the patients in previously reported cases required a blood transfusion. Similar to that, our patient was improved with antibiotic therapy (rifampicin with doxycycline) with corticosteroids. Only one report in the literature described the use of rituximab in a patient of brucellosis with severe hemolysis not responding to corticosteroids [8]. In their case, hepatic and splenic granulomas were present with sacral bone localisation of the organism which probably suggests disseminated nature of the disease and required aggressive immunosuppressive treatment with rituximab. According to the literature, none of the previously reported patients required long-term immunosuppression. Our patient is doing well at 3 months of follow-up and corticosteroids tapered successfully.

In conclusion, brucellosis can present with severe hematological complications like microangiopathic hemolytic anemia (MAHA), Coombs-positive hemolytic anemia, and thrombocytopenia, which may require immunosuppressive therapy along with antibiotics. The prognosis of hematological disease in Brucella infection is relatively good without any relapse. Although rare, brucellosis should be considered as a differential diagnosis in a patient presenting with hemolytic anemia, especially in endemic areas like India.

## Figures and Tables

**Table 1:** Clinical and demographic characteristics of reported hemolytic complications in brucellosis.

First author; year of publication	Country	Number of cases	Age/gender	Hematological disease	Treatment
Kiki et al. [6]; 2008	Turkey	1	19 yr, female	TTP	Doxycycline, rifampicin, plasma exchange
Wehbe and Moore [7]; 2008	United States	1	34 yr, female	AIHA	No immunosuppressant
Sari et al. [10]; 2008	Turkey	1	26 yr, female	AIHA	Corticosteroids
Bourantas et al. [8]; 2010	Greece	1	79 yr, female	AIHA	Corticosteroids, rituximab
Apa et al. [11]; 2013	Turkey	1	5-month infant	AIHA	Rifampicin, trimethoprim/sulfamethoxazole Blood transfusion, corticosteroids
Eskazan et al. [9]; 2014	Turkey	2	72 yr, male; 50 yr, female	AIHA	Doxycycline, rifampicin, corticosteroids
Tunccan et al. [4]; 2014	Turkey	1	75 yr, male	ITP	Doxycycline, rifampicin, corticosteroids
Makis et al. [3]; 2017	Greece	1	5.5 yr female	ITP	Rifampicin, trimethoprim/sulfamethoxazole (TMP/SMX), IV IG

AIHA = autoimmune hemolytic anemia; TTP = thrombotic thrombocytopenic purpura; ITP = immune thrombocytopenic purpura.
